# PHYSICAL AND EMOTIONAL WELL-BEING AMONG OLDER TRANSGENDER AND SEXUAL MINORITY ADULTS

**DOI:** 10.1093/geroni/igad104.2572

**Published:** 2023-12-21

**Authors:** Laura Bernstein, Julie Hicks Patrick

**Affiliations:** West Virginia University, Morgantown, West Virginia, United States; West Virginia University, Morgantown, West Virginia, United States

## Abstract

Health disparities exist between heterosexual adults and those in the LGBTQ+ community. These differences may be exacerbated in late life. However, a comparative approach that includes heterosexual adults as a referent group may obscure important within-group differences among sexual and gender minorities. The purpose of the current study was to examine within-group differences in health and well-being among adults ages 65 and over who identified as LGBTQ+. Data are provided by 2673 adults ages 65+ who completed the Sexual Orientation/Gender Identity module of the 2020 CDC Behavior Risk Factor Surveillance System. Age groups included 1020 young-old adults ages 65 to 70 years, 1056 older adults, ages 71 to 79 years, and 597 old-old adults age 80+. Adults identifying as gay/lesbian (n = 856), bisexual (n=675), transgender heterosexual (n = 135) or some other minoritized sexual identity (n = 1007) supplied assessments of physical, mental, and functional health. A series of 3 (age) by 4 (sexual orientation) ANOVAs examined main effects of age, sexual orientation, and their interaction. Age effects, but not sexual orientation effects, emerged for mental health. Significant effects for functional ability emerged for sexual orientation and for gender, although no interaction was observed. No significant effects emerged for physical health and no interactions emerged as significant for any of the health outcomes. These results emphasize that examining within-group heterogeneity among sexual minority status and age is necessary to localize and address potential health disparities.

